# Visually Induced Motion Sickness During Smartphone Use in Moving Metro Carriages: Effects of Posture and Viewing Duration—A Randomized Crossover Study

**DOI:** 10.3390/healthcare14121707

**Published:** 2026-06-15

**Authors:** Yi-Lang Chen, Chun-Yu Chan, Yun-Pei Fan, Tzu-Ting Wei

**Affiliations:** Department of Industrial Engineering and Management, Ming Chi University of Technology, New Taipei 243303, Taiwan; u12217130@mail2.mcut.edu.tw (C.-Y.C.); u12217112@mail2.mcut.edu.tw (Y.-P.F.); u12217139@mail2.mcut.edu.tw (T.-T.W.)

**Keywords:** MRT carriages, smartphone use, visually induced motion sickness, critical flicker fusion frequency (CFF), visual fatigue scale (VFS), viewing distance

## Abstract

Background/Objectives: Smartphone use during public transit is widespread, yet the combined effects of posture, viewing duration, and sex on visual fatigue and visually induced motion sickness (VIMS) under real commuting conditions remain insufficiently understood. This study examined these factors during smartphone video viewing aboard Taipei MRT carriages. Methods: Forty healthy young adults (20 males, 20 females) completed four randomized conditions crossing two postures (sitting, standing) and two viewing durations (15 min, 30 min). Conditions were administered in a randomized order. Critical flicker fusion frequency (CFF), visual fatigue scale (VFS) scores, viewing distance, and VIMSSQ-short change scores were assessed as primary outcomes and analyzed using three-way mixed ANOVA. Results: Viewing duration produced the largest and most consistent effects across all outcomes (η^2^p = 0.658–0.969): 30 min viewing yielded greater CFF reduction, higher VFS scores, shorter viewing distance, and elevated VIMS compared with 15 min viewing. Standing posture significantly increased VFS scores, shortened viewing distance, and raised VIMS relative to sitting, though its effect on CFF reduction was not statistically significant. A significant sex × viewing duration interaction emerged with regard to VFS scores, with females showing a steeper increase in subjective fatigue over time, despite no significant sex main effect for any outcome. A significant posture × viewing duration interaction for VIMS indicated that standing was associated with greater VIMS responses during prolonged viewing. Conclusions: Prolonged viewing duration and standing posture are key contributors to smartphone-related visual and motion discomfort in metro environments. Limiting continuous viewing time and preferring a seated posture when using smartphones during commuting are recommended strategies to reduce both visual fatigue and VIMS among healthy young adults; generalizability to broader commuter populations warrants further investigation.

## 1. Introduction

Smartphone use has become a routine part of daily commuting, particularly when passengers engage in visually demanding tasks such as video viewing, gaming, and reading. Recent field observations indicate that mobile device engagement is especially prevalent in public transportation systems, where users commonly perform visually intensive tasks such as video viewing and gaming [1,2,3], with postural and behavioral patterns during such use varying considerably across commuters [4,5]. The ubiquity of such behavior has prompted increasing concern regarding the physiological and perceptual consequences of smartphone use under real-world conditions [6].

Digital eye strain can be categorized into external and internal symptoms, including external symptoms related to the ocular surface and internal symptoms arising from the refractive and binocular vision systems [7]. Ergonomic studies on smartphone use typically focus on internal symptoms, such as visual fatigue and headaches, which result from strain on the eye’s refractive and binocular vision systems. Visual fatigue represents one of the most consistently reported outcomes associated with prolonged digital display exposure. From a neuro-optical perspective, sustained near viewing requires continuous coordination between accommodation and vergence systems to maintain binocular clarity, thereby imposing metabolic and neural demands on ocular control mechanisms. Empirical evidence indicates that shorter viewing distances, longer exposure durations, and increased task complexity significantly elevate visual load and fatigue risk [8,9]. Objective indicators such as critical flicker fusion frequency (CFF) have been shown to decrease following prolonged visual tasks, reflecting neural fatigue, whereas subjective ratings capture perceptual discomfort [10,11]. Together, these findings suggest that visual fatigue is a multidimensional response integrating physiological strain and perceptual experience [12,13,14].

Environmental context may further modulate these responses. Unlike laboratory settings, real-world smartphone use often occurs under dynamic conditions that require simultaneous sensory integration and postural control [3,5]. Smartphone interaction during walking has been shown to produce greater fatigue than stationary use [15], indicating that environmental motion can amplify visual demand. Such findings align with theoretical models proposing that cognitive–perceptual load increases when visual processing must be maintained alongside motor stabilization and spatial orientation demands.

In addition to visual fatigue, motion-related responses may play a critical role in determining user comfort in mobile environments. Viewing dynamic content, such as films, has been reported to significantly increase visual fatigue compared to static content [16]. Visually induced motion sickness (VIMS) arises when visual signals conflict with vestibular and proprioceptive inputs, producing symptoms such as dizziness, nausea, and disorientation. Experimental evidence demonstrates that susceptibility to VIMS correlates with real-world motion sickness during visual task performance [17], while neurophysiological studies show that VIMS states are associated with measurable changes in brain connectivity patterns [18]. Even subtle sensory inconsistencies can provoke discomfort during prolonged exposure, highlighting the sensitivity of multisensory integration systems to environmental perturbations [19]. These findings suggest that visually demanding tasks performed in moving environments may simultaneously challenge visual, vestibular, and cognitive systems.

Despite growing recognition of these issues, existing smartphone research has largely examined visual fatigue, musculoskeletal strain, or motion sickness independently. Integrated investigations that simultaneously quantify behavioral viewing characteristics, objective fatigue indices, subjective symptoms, and motion-related responses remain scarce, particularly under naturalistic transportation conditions. This limitation constrains understanding of how multiple physiological systems interact during real-world device use.

A previous field study demonstrated that posture and viewing duration significantly influence visual fatigue during smartphone viewing in MRT environments [20]; however, that investigation did not assess motion-related symptoms, leaving unresolved whether visually induced motion sickness contributes to overall user strain in dynamic transit contexts. Given that commuters commonly engage with handheld displays while exposed to vehicle motion, evaluating both visual and motion-related responses is essential for understanding the full physiological impact of smartphone use. The two viewing durations selected for this study—15 and 30 min—were chosen to represent short-hop commuting segments and the average one-way commuting time in Taipei (approximately 32.7 min), respectively, and are consistent with durations used in prior smartphone viewing protocols [21]. CFF was selected as the primary objective outcome measure because it is a well-validated, sensitive, and non-invasive index of neural visual fatigue widely used in digital display research [22,23]. Therefore, the present study aimed to examine the combined effects of viewing posture (sitting vs. standing), viewing duration (15 vs. 30 min), and sex on visual fatigue, viewing distance, and VIMS during smartphone video viewing aboard Taipei MRT carriages, using both objective (CFF reduction) and subjective (VFS, VIMSSQ-short) outcome measures. The findings are intended to provide ecologically valid evidence for ergonomic guidance on mobile device use in public transportation environments.

## 2. Materials and Methods

The following subsections describe the participant characteristics, measurement instruments, experimental protocol, and statistical approach used in this study. To assess visual load and VIMS during smartphone video viewing in MRT carriages, 40 participants engaged in smartphone video-viewing trials for either 15 or 30 min while adopting either a sitting or standing posture. Data collection included CFF reduction, self-reported VFS scores, viewing distance, and VIMS responses. These metrics were employed to evaluate the impact of mobile video viewing on visual fatigue, viewing behavior, and motion-related discomfort. This study received ethical approval from the Ethics Committee of National Taiwan University in Taiwan (Approval Code: NTU-REC 202312EM051), affirming its adherence to ethical standards and guidelines. All testing processes were carried out in accordance with the relevant guidelines and regulations of the 2013 World Medical Association Declaration of Helsinki. Written informed consent was obtained from all participants, including consent for publication of any identifiable images.

### 2.1. Participants

In this study, we recruited 40 individuals, equally divided by sex, with no history of musculoskeletal disorders or visual impairments. The selection criteria required a minimum of one year of daily smartphone usage for at least 3 h, based on prior research. Among male participants, the average (±standard deviation) age, height, and body mass were 20.4 (±0.5) years, 174.3 (±4.8) cm, and 71.3 (±11.7) kg, respectively. Female participants had an average age, height, and body mass of 20.7 (±0.6) years, 158.6 (±5.7) cm, and 55.8 (±7.9) kg, as detailed in Table 1. Notably, the male participants were significantly taller and heavier than the female participants (*p* < 0.001). However, baseline CFF values were comparable between sexes, averaging 39.8 Hz for males and 39.5 Hz for females. This CFF range aligned with reported typical values for adults, which fall between 35 and 40 Hz [24]. Prior to data collection, participants received a thorough explanation of the testing protocol and provided informed consent using a consent form. Prior to enrollment, participants were also asked about any history of dizziness, vertigo, chronic motion sickness, or known vestibular conditions; individuals reporting such history were excluded.

### 2.2. The CFF Measurement

CFF is a well-recognized metric used to assess visual fatigue, where a reduction in CFF value corresponds to increased eye fatigue [22,23]. We measured CFF using a Handy Flicker device (Handy Flicker HF-II, Neitz, Tokyo, Japan). The ascending and descending thresholds were systematically recorded and then averaged to establish the pre- and post-task CFF for each measurement. This process of measuring CFF was repeated twice, and the resulting values were averaged for precision. CFF reduction was calculated as pre-task CFF minus post-task CFF; therefore, larger positive values indicated greater visual fatigue.

During each test, the flicker frequency was systematically increased from a minimum threshold of 20 Hz until participants consistently perceived the presented light stimuli as stable. This threshold represented the frequency at which participants no longer perceived the stimulus as flickering. Following this, the frequency was gradually decreased until participants indicated that they once again perceived the presented light stimuli as flickering or vibrating, following the methodology outlined by Gautam and Vinay [24].

### 2.3. VIMS Measurement

VIMS was quantified using the short form of the Visually Induced Motion Sickness Susceptibility Questionnaire (VIMSSQ-short), a brief, validated self-report instrument developed specifically for discomfort associated with visual technologies and displays [25]. The VIMSSQ-short comprises six items assessing the frequency/severity of core VIMS-related symptoms—nausea, headache, fatigue, dizziness, and eye strain—plus a behavioral consequence item reflecting avoidance/urge to stop viewing. Items are rated on a 4-point scale from 0 (none) to 3 (severe), and summed to produce a total score ranging from 0 to 18, with higher scores indicating greater VIMS.

Participants completed the VIMSSQ-short immediately before and immediately after each experimental condition (posture × viewing duration). To capture condition-evoked symptom changes while controlling for baseline variability, we computed a VIMS increase score for each condition as post-exposure total minus pre-exposure total. This change score served as the dependent variable in the subsequent three-way mixed ANOVA (Sex as a between-subject factor; Posture and Viewing duration as within-subject factors).

The VIMSSQ-short was selected because it is explicitly designed for visually induced motion sickness contexts (including smartphone viewing) and is suitable for repeated administration due to its brevity, minimizing respondent burden and reducing carryover fatigue across conditions. In large-sample normative work, the VIMSSQ-short demonstrates good internal consistency (reported Cronbach’s α was approximately 0.80), supporting its reliability for quantifying individual differences and condition-related symptom changes. The questionnaire was administered in Traditional Chinese. As no validated Traditional Chinese version is currently available in the literature, a forward translation was prepared by the research team and independently reviewed by two bilingual researchers with expertise in ergonomics to assess semantic consistency with the original English version. Internal consistency of the translated version in the present sample was acceptable (Cronbach’s α = 0.80), consistent with values reported in the original validation study.

To standardize reporting and reduce demand characteristics, participants were instructed to base their responses on symptoms during the immediately preceding trial and to answer all items independently. Questionnaires were checked for missing responses on-site; if any item was omitted, the participant was prompted to complete it before leaving. If an item remained missing, the total score for that condition was treated as missing and excluded from VIMS analyses. This approach ensured consistent scoring across conditions while maintaining transparency in data handling.

### 2.4. Subjective Visual Fatigue Rating

In this study, the VFS score was used to assess eye strain after viewing the films in a requested testing condition [26]. The VFS questionnaire comprises six items: (1) It is hard for me to see; (2) I have a strange feeling around my eyes; (3) My eyes feel tired; (4) I feel numb; (5) I feel dizzy looking at the screen; (6) I have a headache. Participants rated these questions on a 10-point scale, where 1 indicated “Not at all” and 10 indicated “Extremely serious”. The scores for the six items were then averaged to provide an overview of the severity of experienced visual fatigue. In our analysis, we considered the change in the VFS resulting from the undertaken film viewing activity. Specifically, the fatigue score recorded at the beginning of each testing session was regarded as the baseline against which subsequent measurements were compared.

### 2.5. The VD Measurements

**Figure 1:**
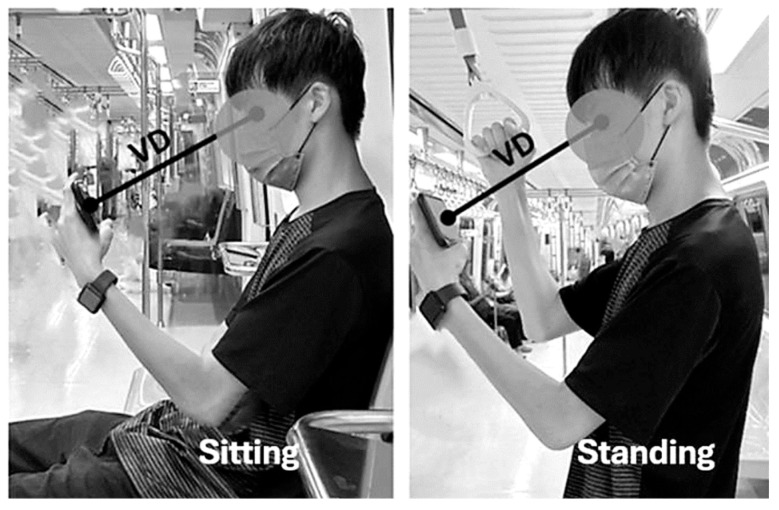
Measurement of viewing distance (VD) during standing and sitting smartphone use.

Since this study was conducted in actual MRT carriages, it lacked the controlled environment of a laboratory. To mitigate this, we pre-selected a fixed position in the carriage for conducting the sitting and standing experiments. During the pilot test, we positioned a camera 2 m away from the vertical sagittal plane of the participant. We then used the camera’s controlled parameters to establish the dimensional ratio on the participant’s sagittal plane (Figure 1). After calibration, this ratio was employed as the basis for measuring the VD during the experiments, estimating the actual VD from the size obtained on the image. Previous studies consistently show that shorter VDs increase the strain on accommodation and vergence, potentially exacerbating eye strain symptoms [9,27,28,29,30]. To derive VD, we meticulously captured symmetrical sagittal images and utilized CorelDRAW (Corel Co., Ottawa, ON, Canada, Graphics Suite, 2025) for precise digital markings. The experimenters identified the participant’s eyeball and the midpoint of the phone’s length on the digital images to calculate VD. In the pilot test, the discrepancy between actual and estimated VD was merely 0.6 cm, confirming acceptable measurement accuracy.

### 2.6. Experimental Design and Procedure

This study collected data on CFF reduction, VFS scores, VD measurements, and VIMS responses across four distinct smartphone-viewing trials. These trials combined two viewing postures (sitting and standing) and two viewing durations (15 min and 30 min) for 40 young participants. The 30 min viewing duration aligns with a prior study in which participants watched a movie on a smartphone while walking on a treadmill or sitting in a chair [21], though intermediate times were not measured. Given that commuters in Taipei spend approximately 1 h daily in transit [31], the selected durations are representative of typical exposure windows. In each trial, CFF and VFS assessments were conducted at the start and end of the viewing session. Participants watched four films in four different combinations of posture and duration, each performed in separate sessions to avoid accumulating fatigue. These combinations were arranged in a randomized sequence. Participants used their own smartphones with the assigned movies pre-loaded before the experiments. To avoid distorting the testing situation compared to the real world, participants were allowed to carry their usual commuting backpacks. However, backpacks that were too large or heavy, potentially altering body posture, were excluded from the study. Each trial lasted 15 or 30 min, with VD data recorded during the final 1 min interval at 15 s intervals. These values were averaged for analysis across four time points to reduce the influence of transient postural deviations, including lateral sway during standing. To minimize errors and participant fatigue, a minimum 10 min resting period was imposed between trials. The Taipei MRT carriage typically maintains a temperature of 24 °C, with an average speed of 35 km/h and a maximum speed of 80 km/h. The average illumination in the carriage at 100 cm above the floor is over 250 lx, with a minimum of 200 lx [32]. All testing sessions for a given participant were conducted at the same time of day on the same MRT line and route, minimizing circadian and route-related variability across conditions.

### 2.7. Statistical Analysis

The data collected from the study underwent thorough analysis using SPSS 23.0 statistical software (IBM Corp., Armonk, NY, USA), with a significance level of 0.05 for all tests. The primary objective was to examine the effects of participant sex, viewing posture (sitting and standing), and viewing duration (15 min and 30 min) on CFF reduction, VFS change score, VD, and VIMS increase score using three-way mixed ANOVA. In the analysis, participant sex was designated as a between-subject factor, while posture and duration were considered within-subject factors. Where significant interactions were observed, simple-effects analyses were conducted. Paired-sample comparisons were used for within-subject effects, whereas independent-sample comparisons were used for between-sex comparisons. To assess the practical significance of any identified independent variable, effect sizes were reported as partial eta squared (η^2^p) for ANOVA effects [33]. Values of approximately 0.01, 0.06, and 0.14 were interpreted as small, medium, and large effects, respectively. Prior to conducting the analyses, the Shapiro–Wilk test was utilized to evaluate the alignment of numerical variables with the normal distribution. Additionally, Levene’s test was employed to examine the equality of variances, ensuring the robustness of the analytical framework. In addition, repeated-measures correlations (rmcorr) were computed across all 160 observations (40 participants × 4 conditions) to explore associations among CFF reduction, VFS change score, viewing distance, and VIMS increase score.

## 3. Results

### 3.1. Preliminary Checks

All outcome variables satisfied the normality assumption (Shapiro–Wilk tests, all *p* > 0.05) and homogeneity of variance (Levene’s tests, all *p* > 0.05). Baseline CFF did not differ between sexes (males: 39.8 ± 2.9 Hz; females: 39.5 ± 2.3 Hz; *p* = 0.719), confirming comparable visual status at the start of each session. For the VIMS increase score specifically, skewness was 0.22 and excess kurtosis was 0.02, indicating a symmetric, mesokurtic distribution with no evidence of floor-related skew (Shapiro–Wilk *p* = 0.837).

### 3.2. CFF Reduction

**Figure 2:**
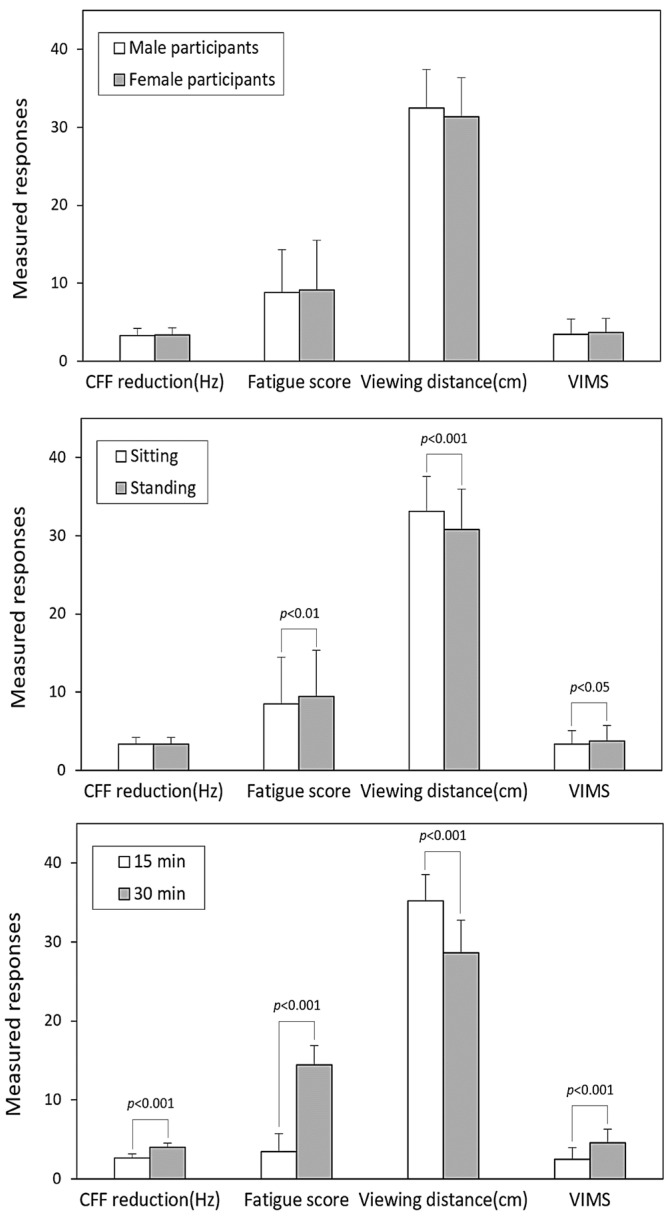
Main effects of sex, posture, and viewing duration on CFF reduction, visual fatigue score, viewing distance, and VIMS increase score during smartphone viewing in MRT carriages. Error bars indicate standard errors. CFF = critical flicker fusion frequency; VIMS = visually induced motion sickness increase score.

Viewing duration exerted a dominant effect on CFF reduction (*p* < 0.001, η^2^p = 0.938), with the 30 min condition producing substantially greater CFF decline than the 15 min condition (Figure 2). Mean CFF reduction was 3.69 ± 0.03 Hz at 30 min compared with 3.00 ± 0.03 Hz at 15 min, representing an increase of approximately 23% in objective visual fatigue with doubling of exposure time. Neither posture (*p* = 0.960) nor sex (*p* = 0.424) reached significance, and no interaction terms were significant (all *p* > 0.05; Table 2).

### 3.3. Subjective Visual Fatigue (VFS)

**Figure 3:**
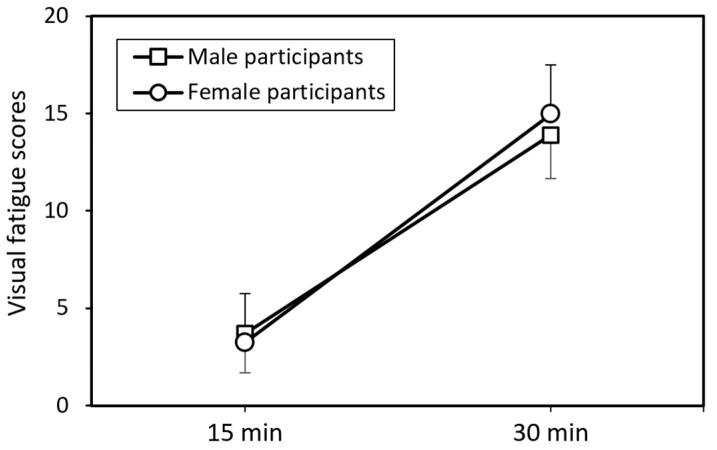
Interaction of sex and viewing duration on visual fatigue change scores.

Both viewing duration (*p* < 0.001, η^2^p = 0.969) and posture (*p* = 0.003, η^2^p = 0.213) significantly increased VFS scores, with 30 min viewing and standing posture each associated with higher fatigue ratings (Figure 2). Mean VFS scores were 11.70 ± 0.13 at 30 min versus 6.15 ± 0.12 at 15 min, and 9.13 ± 0.33 during standing versus 8.72 ± 0.34 during sitting. Sex as a main effect was non-significant (*p* = 0.489). A significant sex × viewing duration interaction was observed (*p* = 0.020, η^2^p = 0.134), indicating that females reported a steeper rise in subjective fatigue from 15 to 30 min compared with males (Figure 3). Specifically, females increased from 6.03 ± 0.20 at 15 min to 11.93 ± 0.19 at 30 min (a rise of 5.90 points), whereas males increased from 6.28 ± 0.15 to 11.46 ± 0.16 (a rise of 5.18 points). All other interactions were non-significant (Table 2).

### 3.4. Visually Induced Motion Sickness (VIMS)

**Figure 4:**
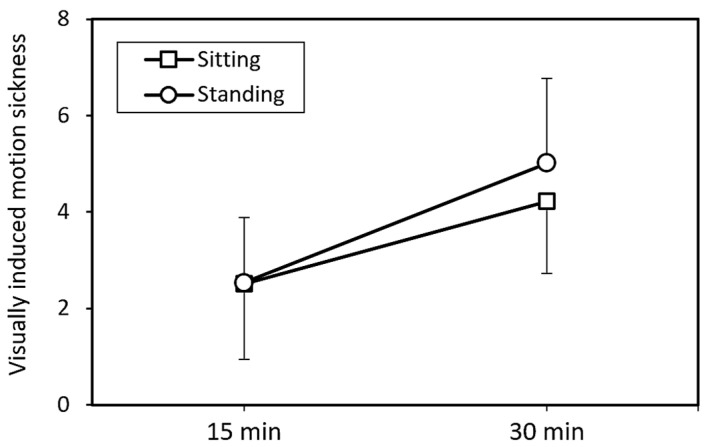
Interaction of posture and viewing duration on visually induced motion sickness (VIMS) increase score.

Viewing duration showed the largest effect on VIMS increase score (*p* < 0.001, η^2^p = 0.658), followed by posture (*p* = 0.039, η^2^p = 0.107), with greater symptoms after 30 min viewing and in the standing condition. Mean VIMS scores were 4.09 ± 0.09 at 30 min versus 3.06 ± 0.08 at 15 min, and 3.68 ± 0.11 during standing versus 3.47 ± 0.10 during sitting. A significant posture × viewing duration interaction was also detected (*p* = 0.041, η^2^p = 0.105), suggesting that the additional VIMS burden of standing was more pronounced at 30 min than at 15 min (Figure 4). In particular, the standing condition at 30 min yielded the highest VIMS score (4.28 ± 0.13), compared with sitting at 30 min (3.91 ± 0.12), standing at 15 min (3.08 ± 0.11), and sitting at 15 min (3.04 ± 0.12). Sex main effect and all other interaction terms were non-significant (all *p* > 0.05; Table 2).

### 3.5. Inter-Variable Correlations

Repeated-measures correlations (rmcorr) computed across all 160 observations (Table 3) revealed moderate-to-strong associations among outcome variables. These correlations represent within-participant associations after controlling for between-participant variability. CFF reduction correlated strongly with VFS score (r = 0.839) and moderately with VIMS (r = 0.652). CFF reduction was also negatively correlated with viewing distance (r = −0.677). Viewing distance correlated inversely with both VFS score (r = −0.766) and VIMS (r = −0.527), indicating that shorter distances co-occurred with greater fatigue and motion sickness. The correlation between VFS and VIMS was also significant (r = 0.698). All inter-variable correlations were statistically significant (all *p* < 0.001).

## 4. Discussion

The present study examined visual fatigue, viewing behavior, and visually induced motion sickness (VIMS) during smartphone video viewing in a real MRT carriage. The main findings were clear and consistent. Viewing duration showed the largest and most consistent effects across outcomes, including CFF reduction, subjective visual fatigue, viewing distance, and VIMS. Standing posture further increased subjective fatigue and VIMS and shortened viewing distance, whereas sex showed no main effect on any outcome. Two interaction patterns were also noteworthy: subjective fatigue increased more steeply from 15 to 30 min in females, and the additional VIMS burden of standing was more evident after 30 min of viewing. Overall, these findings indicate that both viewing duration and posture contributed to the observed discomfort responses during smartphone use in moving public transportation.

### 4.1. Effects of Viewing Duration on Visual Fatigue

The strong effect of viewing duration on CFF reduction and VFS scores supports the view that sustained near-screen viewing is a major source of visual fatigue. Prolonged smartphone viewing requires continuous accommodation and vergence control, and these demands may be associated with progressively greater ocular and neural load. This interpretation is consistent with previous studies showing that digital display use can increase subjective eye symptoms and reduce visual performance indicators after sustained exposure [12,13,14]. It also agrees with reviews and empirical work linking digital eye strain to longer exposure time, near viewing, and accommodative stress [8,29,34,35].

The present CFF findings are particularly important because CFF reduction has been widely used as an objective index of visual fatigue or workload [22,23]. Although recent studies have cautioned that CFF may not fully capture all dimensions of digital eye strain [10,11], the current results showed that CFF reduction and subjective fatigue were strongly correlated. This suggests that, in the present MRT viewing task, objective and subjective indicators may reflect a common time-dependent fatigue process. The finding extends an earlier MRT study [20] that focused primarily on eye strain by showing that the duration effect remains robust even when VIMS is assessed simultaneously.

### 4.2. Effects of Posture and Viewing Distance

Standing posture significantly increased subjective fatigue and shortened viewing distance, although it did not significantly affect CFF reduction. This pattern suggests that posture may influence perceived discomfort and viewing behavior more strongly than the specific neural fatigue component captured by CFF. In a moving metro carriage, standing requires continuous balance adjustments and postural stabilization. These additional demands may increase attentional and sensorimotor load while users attempt to maintain stable fixation on a handheld display. This explanation is consistent with research showing that smartphone use in mobile or unstable contexts can impose greater physiological and perceptual strain than stationary use [15,21].

The shorter viewing distances observed during standing and after longer viewing are also ergonomically meaningful. Prior studies have reported that handheld smartphone use often occurs at close viewing distances, which increases accommodative and vergence demand and may contribute to visual discomfort [9,27,28]. In the present study, viewing distance was negatively correlated with both VFS and VIMS, indicating that shorter distances tended to co-occur with greater discomfort. However, these correlations should not be interpreted as proving a direct causal pathway. Users may shorten viewing distance as an adaptive response to carriage movement, small screen content, or reduced postural stability, and this behavior may then further increase visual load. Thus, viewing distance appears to be both an ergonomic exposure factor and a behavioral indicator of adaptation during mobile device use. The reduction in viewing distance during standing may also partly reflect a biomechanical stabilization strategy, whereby users hold the device closer to reduce the fine motor demands of stabilizing a handheld display while simultaneously managing dynamic postural balance in a moving carriage.

### 4.3. VIMS Responses in a Moving Metro Environment

A key contribution of this study is the incorporation of VIMS assessment into a real-world smartphone-viewing paradigm. VIMS increased significantly with longer viewing duration and standing posture, indicating that smartphone use in transit may involve not only visual fatigue but also motion-related discomfort. According to the sensory conflict theory, motion sickness occurs when visual, vestibular, and proprioceptive signals provide incongruent information about self-motion [36]. In an MRT carriage, the body receives acceleration and balance-related cues from vehicle motion, whereas the smartphone screen provides a relatively stable near-field visual target. This sensory mismatch may be amplified during standing, although the underlying mechanisms were not directly measured in the present study.

The posture × viewing duration interaction for VIMS further indicates that the standing condition was associated with greater VIMS responses when viewing was prolonged. This finding is consistent with experimental and review evidence showing that visually demanding tasks and longer exposure durations can increase motion-sickness-like symptoms in vehicles or visually dynamic environments [4,37,38]. It is also compatible with recent evidence that VIMS susceptibility is related to on-road car sickness during visual task performance [17]. The present results extend these findings to daily metro commuting, where motion exposure is less controlled but more ecologically valid than most simulator or virtual-reality protocols. It should also be noted that substantial interindividual variability in VIMS susceptibility was evident, as reflected in the large standard errors visible in Figure 4. Such variability is consistent with prior evidence that VIMS susceptibility differs considerably across individuals [38,39], and future studies should consider stratifying participants by baseline susceptibility or including it as a covariate.

### 4.4. Relationship Between Visual Fatigue and VIMS

The correlation analysis showed that VFS scores were moderately associated with VIMS, while CFF reduction was also significantly associated with VIMS. These relationships indicate partial overlap between visual fatigue and motion-related symptoms. Such overlap is reasonable because several VIMSSQ-short items, including headache, fatigue, dizziness, and eye strain, may reflect broader perceptual and autonomic discomfort during screen viewing [25]. At the same time, the correlations were not so high as to suggest that the two constructs are identical. Visual fatigue is mainly related to sustained ocular and visual processing demands, whereas VIMS also involves multisensory integration, vestibular conflict, and autonomic responses. Therefore, evaluating both visual fatigue and VIMS provides a more complete understanding of smartphone-related discomfort in moving environments than either measure alone.

This integrated interpretation is consistent with neurophysiological and human factor evidence suggesting that VIMS involves central sensory processing rather than only visual stimulation. For example, VIMS has been associated with changes in functional connectivity during visually provocative exposure [18], and even subtle visual instability can induce discomfort in visually mediated environments [19]. In the current MRT setting, the combined effects of prolonged viewing, close viewing distance, and vehicle motion may have increased both visual workload and multisensory conflict. This may help explain why duration and posture were associated with changes in both fatigue and VIMS outcomes.

### 4.5. Sex Differences and Interaction Effects

Sex did not show significant main effects on CFF reduction, VFS score, viewing distance, or VIMS. This finding differs from some motion sickness literature reporting greater susceptibility among females [37], but it may be explained by the relatively moderate and naturalistic motion exposure in the present study. Unlike highly provocative virtual-reality or simulator conditions, MRT travel involves variable but generally tolerable motion levels. Under such conditions, posture and exposure duration may dominate the response, while sex-related susceptibility may be less apparent.

Nevertheless, the significant sex × viewing duration interaction for subjective visual fatigue suggests that females experienced a greater increase in perceived fatigue from 15 to 30 min. Because baseline CFF did not differ between sexes, this interaction is unlikely to reflect substantial initial differences in visual status. Instead, it may indicate a difference in cumulative symptom perception, tolerance, or reporting over prolonged exposure. This finding should be interpreted cautiously because the interaction appeared only for VFS and not for CFF, viewing distance, or VIMS. Future studies with larger samples and additional physiological measures may help clarify whether this reflects a stable sex-related response pattern or context-specific variability. It is also worth considering whether repeated exposure across the four conditions may have introduced some degree of motion adaptation or habituation, which could have moderated VIMS responses in later conditions. Although full randomization was used to distribute such effects across conditions, adaptation cannot be entirely ruled out in a within-subject design. Future studies employing between-subject designs or longer inter-session intervals could help clarify the extent to which habituation contributes to the observed pattern of results.

### 4.6. Practical Implications

Based on the present findings in healthy young adults, the following tentative implications may be offered for commuter behavior and ergonomic guidance. First, limiting continuous smartphone viewing time appears to be the most practical strategy for reducing both visual fatigue and VIMS, because duration showed the largest effects across outcomes. Second, using smartphones while seated may reduce subjective fatigue, help maintain a longer viewing distance, and lower VIMS compared with standing. Third, users should be encouraged to maintain an adequate viewing distance and to take short visual breaks during longer commutes. These recommendations are consistent with general digital eye strain prevention strategies and are particularly relevant for public transportation, where environmental motion and postural instability cannot be fully controlled. It should be noted, however, that these suggestions are based on a sample of healthy young adults under specific commuting conditions, and their applicability to other populations and settings requires further validation.

For device and interface design, the results suggest that mobile content intended for transit use should minimize unnecessary visual demand. Larger text, stable layouts, reduced visual motion, and features that encourage periodic breaks may be useful for reducing discomfort. Because video viewing and gaming are common in public transportation settings [1,2], interface designs that reduce close fixation and rapid visual motion may be especially important for commuters who must stand during travel. More broadly, habitual smartphone use during commuting may contribute to cumulative sedentary behavior patterns linked to metabolic health risks, particularly among young adults who rely heavily on public transit for daily mobility [40].

### 4.7. Limitations and Future Research

Several limitations should be noted. First, the real MRT environment increased ecological validity but also introduced uncontrolled variability, including carriage acceleration, vibration, crowding, lighting, and route-specific motion profiles. These factors may have contributed to variability in VIMS and viewing behavior. Although participants were screened through self-report for a history of dizziness, vertigo, chronic motion sickness, and known vestibular disorders, no standardized clinical vestibular assessment was conducted. Consequently, undetected subclinical vestibular sensitivities may have contributed to interindividual variability in VIMS responses. Furthermore, acceleration, deceleration, and lateral sway were not directly quantified during individual testing windows; therefore, participants may have experienced different motion exposures depending on train dynamics at the time of testing. Future studies could combine field testing with synchronized acceleration, vibration, postural sway, and wearable accelerometer measurements to better characterize the mechanical sources of discomfort and motion-related symptoms.

Second, no a priori power analysis was conducted; this is acknowledged as a limitation of the present study. The sample size of 40 was informed by prior related research, and the large observed effect sizes for duration effects (η^2^p ≥ 0.65) suggest adequate power for primary main effects, though interaction effects should be interpreted with appropriate caution. Additionally, the four experimental conditions were assigned in a randomized order rather than formally counterbalanced (e.g., Latin square), which may have resulted in uneven exposure to order effects across participants. Participants were required to use smartphones for at least three hours daily, which may reduce generalizability to infrequent users. Screen brightness and font size were not standardized across participants, and smartphone screen sizes ranged from approximately 5.0 to 6.5 inches with display resolution uncontrolled; these factors may have introduced variability in visual load across participants.

Third, participants were healthy young adults, which limits generalization to older adults, children, or individuals with visual, vestibular, or balance impairments. Age-related reductions in accommodation, changes in postural control, and differences in motion sensitivity may alter the effects observed here. Fourth, this study examined only two viewing durations. Although 15 and 30 min are relevant to commuting behavior in Taipei, additional time points would help determine whether fatigue and VIMS increase linearly, reach a plateau, or show adaptation over longer exposures. Fifth, VIMS susceptibility was not stratified before testing. Because individual differences in motion sickness susceptibility are substantial [37,39], future research should consider including baseline susceptibility measures as covariates or grouping variables.

Finally, although CFF, VFS, viewing distance, and VIMSSQ-short scores provided complementary information, the study did not include eye-tracking, blink rate, accommodation response, or physiological indices of autonomic arousal. Incorporating these measures would allow more precise identification of the pathways linking visual fatigue, viewing behavior, postural demand, and motion-related discomfort.

## 5. Conclusions

This study provides real-world evidence that smartphone viewing in moving MRT carriages can elicit both visual fatigue and visually induced motion sickness, and that these responses are mainly shaped by viewing duration and posture. Prolonged viewing produced the clearest effect, particularly for CFF reduction, subjective visual fatigue, viewing distance, and VIMS increase score, indicating that cumulative exposure is a central contributor to discomfort during mobile screen use. Standing posture further increased subjective fatigue, shortened viewing distance, and elevated VIMS, with the posture-related VIMS burden becoming more evident after 30 min of viewing. By contrast, sex differences were limited; only the increase in subjective fatigue over time differed significantly between males and females. Together, these findings suggest that reducing continuous viewing time and, where possible, using smartphones while seated may help mitigate visual strain and motion-related discomfort during commuting. The results extend previous smartphone eye strain research by integrating objective, subjective, behavioral, and VIMS outcomes in an ecologically valid transit setting, and they provide practical evidence for ergonomic guidance on mobile device use in dynamic transportation environments. It should be acknowledged that these findings are based on a sample of healthy young adults (mean age approximately 20 years), and generalization to broader commuter populations, including older adults, individuals with vestibular sensitivities, or those with varying levels of motion sickness susceptibility, requires further investigation with more diverse samples.

## Figures and Tables

**Table 1:** Basic information of male and female participants in the study.

Items	Males (n = 20)	Females (n = 20)	Differences	*p*
Age (years)	20.4 (0.5)	20.7 (0.6)	−0.3	0.094
Stature (cm)	174.3 (4.8)	158.6 (5.7)	15.7	<0.001
Body mass (kg)	71.3 (11.7)	55.8 (7.9)	15.5	<0.001
CFF (Hz)	39.8 (2.9)	39.5 (2.3)	0.3	0.719

Note: Data (mean, with standard deviation in parentheses) were examined between sexes by independent *t* test. CFF, critical flicker fusion frequency.

**Table 2:** Results of three-way mixed ANOVA examining the effects of sex, posture, and viewing duration on visual fatigue indicators and visually induced motion sickness.

Variables	Responses	MS	F	*p*	η^2^p
Sex (S)	CFF reduction	0.28	0.65	0.424	0.017
	Visual fatigue score	4.26	0.49	0.489	0.013
	Viewing distance	54.14	1.79	0.188	0.045
	VIMS	1.50	0.32	0.572	0.008
Posture (P)	CFF reduction	<0.01	0.00	0.960	<0.001
	Visual fatigue score	36.74	10.29	0.003	0.213
	Viewing distance	218.16	30.50	<0.001	0.445
	VIMS	6.72	4.56	0.039	0.107
Viewing duration (D)	CFF reduction	77.37	570.34	<0.001	0.938
	Visual fatigue score	4814.36	1188.42	<0.001	0.969
	Viewing distance	1710.06	215.23	<0.001	0.850
	VIMS	175.09	73.25	<0.001	0.658
S × P	CFF reduction	0.26	0.99	0.326	0.025
	Visual fatigue score	1.49	0.42	0.522	0.011
	Viewing distance	0.15	0.02	0.886	0.001
	VIMS	0.66	0.45	0.506	0.012
S × D	CFF reduction	0.00	0.00	0.995	0.000
	Visual fatigue score	23.80	5.88	0.020	0.134
	Viewing distance	1.83	0.23	0.634	0.006
	VIMS	0.21	0.09	0.770	0.002
P × D	CFF reduction	0.03	0.12	0.732	0.003
	Visual fatigue score	0.92	0.19	0.666	0.005
	Viewing distance	23.69	3.98	0.053	0.095
	VIMS	5.88	4.47	0.041	0.105
S × P × D	CFF reduction	0.02	0.08	0.778	0.002
	Visual fatigue score	0.10	0.02	0.889	0.001
	Viewing distance	0.36	0.06	0.807	0.002
	VIMS	0.84	0.64	0.428	0.017

Note: all degrees of freedom = 1, 38. η^2^p = partial eta squared. CFF = critical flicker fusion frequency. VIMS = visually induced motion sickness increase score.

**Table 3:** Repeated-measures correlations among outcome variables (n = 160 observations).

Variable Pair	r	95% CI	*p*-Value
CFF reduction vs. Fatigue score	0.839	0.782–0.887	<0.001
CFF reduction vs. Viewing distance	−0.677	−0.762–−0.568	<0.001
CFF reduction vs. VIMS	0.652	0.536–0.741	<0.001
Fatigue score vs. Viewing distance	−0.766	−0.829–−0.680	<0.001
Fatigue score vs. VIMS	0.698	0.585–0.784	<0.001
Viewing distance vs. VIMS	−0.527	−0.640–−0.382	<0.001

Note: CFF = critical flicker fusion frequency; VIMS = visually induced motion sickness increase score. Degree of freedom = 119.

## Data Availability

The data are not publicly available due to ethical restrictions and privacy concerns involving human participants. The data may be available from the corresponding author upon reasonable request and with permission from the Institutional Review Board.

## Abbreviations

The following abbreviations are used in this manuscript:

CFF	Critical flicker fusion frequency
VFS	Visual fatigue scale
VIMS	Visually induced motion sickness
VD	Viewing distance
